# Regulatory Peptides in Asthma

**DOI:** 10.3390/ijms222413656

**Published:** 2021-12-20

**Authors:** Katarzyna Kaczyńska, Dominika Zając, Piotr Wojciechowski, Monika Jampolska

**Affiliations:** Department of Respiration Physiology, Mossakowski Medical Research Institute, Polish Academy of Sciences, Pawińskiego 5 St., 02-106 Warsaw, Poland; dzajac@imdik.pan.pl (D.Z.); pwojciechowski@imdik.pan.pl (P.W.); mjampolska@imdik.pan.pl (M.J.)

**Keywords:** regulatory peptides, asthma, airway hyperreactivity, inflammation

## Abstract

Numerous regulatory peptides play a critical role in the pathogenesis of airway inflammation, airflow obstruction and hyperresponsiveness, which are hallmarks of asthma. Some of them exacerbate asthma symptoms, such as neuropeptide Y and tachykinins, while others have ameliorating properties, such as nociception, neurotensin or β-defensin 2. Interacting with peptide receptors located in the lungs or on immune cells opens up new therapeutic possibilities for the treatment of asthma, especially when it is resistant to available therapies. This article provides a concise review of the most important and current findings regarding the involvement of regulatory peptides in asthma pathology.

## 1. Introduction

Bronchial asthma is a heterogeneous disease characterized by chronic inflammation of the airways, diagnosed on the basis of characteristic symptoms, such as tightness in the chest, wheezing, dyspnea and cough. These symptoms are accompanied by reversible, either spontaneously or by treatment, expiratory airway obstruction. Asthma, regarded as a global health problem, affects more than 300 million people worldwide, but the mechanism of this pathology has not been completely elucidated [1,2].

The inflammatory process in the airway involves a number of cells, e.g., eosinophils, neutrophils, T lymphocytes, macrophages, mast cells and epithelial cells; and many endogenous inflammatory mediators, such as histamine, cysteinyl leukotrienes and cytokines [3].

Interestingly, many other endogenous mediators, such as regulatory peptides not necessarily directly related to respiratory tract functioning, have been implicated in mediation of asthma related inflammatory process and airway hyperreactivity (AHR) [4,5].

**Figure 1:**
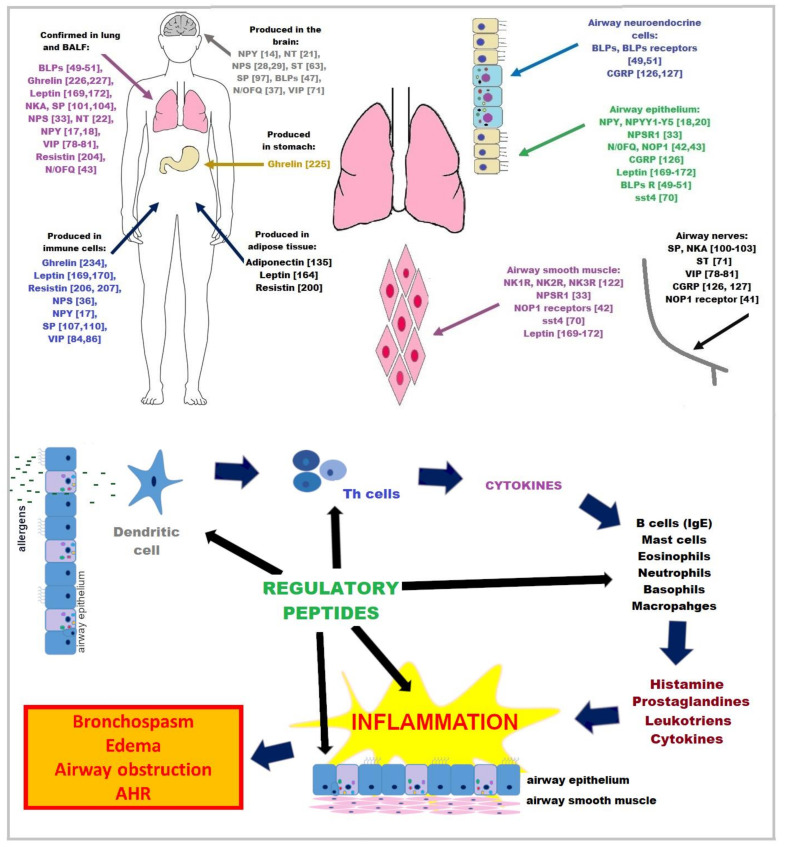
Localization of asthma-related regulatory peptides and their receptors in the body and in the lungs (upper panel). Lower panel shows the locations of regulatory peptide interactions that may influence the course of asthma. NPY, neuropeptide Y; NPS, neuropeptide S; BLPs, bombesin-like peptides; NKA, neurokinin A; SP, substance P; VIP, vasoactive intestinal polypeptide; N/OFQ, nociception; CGRP, calcitonin gene–related peptide.

Some of the regulatory peptides, including neuropeptides, are present in the respiratory system. They are produced by pulmonary neuroendocrine cells (bombesin-like peptides and serotonin), the only innervated airway epithelial cells [6]; and autonomic airway fibers innervating airway muscles, blood vessels and lungs (substance P (SP), neurokinin A (NKA) and vasoactive intestinal polypeptide (VIP)) [5]. Neuropeptides can act directly through their receptors and regulate airway smooth muscle tone, bronchial blood flow and airway secretion or act on inflammatory cells, contributing in this way to inflammatory process and ventilation in asthma pathology (Figure 1) [5,7,8,9]. Their impact on asthma symptoms can be either soothing, as for neurotensin [10], or aggravating, as for neuropeptide Y [11]. Another group of regulatory peptides are adipokines, which are hormones secreted by adipose tissue, namely adiponectin, leptin and resistin; and hormones related to the feeling of hunger and satiety, such as ghrelin. Changes in the physiological balance between proinflammatory and anti-inflammatory adipokines, as well as changes in the physiological balance of adipokines and proinflammatory cytokines secreted by adipose tissue, may increase the risk of obesity-related asthma by reducing the natural development of immune tolerance [12,13].

In this concise review, we summarize major findings, including the recent ones, that are concerned on regulatory peptides involvement in asthma pathology (Table 1). Since successful control of the asthma symptoms is still a challenge, some of the results described may provide helpful guidance in the development of new therapeutic approaches for the treatment of asthma.

## 2. Neuropeptides

### 2.1. Neuropeptide Y (NPY)

Neuropeptide Y (NPY), a 36-amino acid peptide, is the most widespread peptide in neurons of the central and peripheral nervous system, acting through six subtypes of G_i_/G_o_-coupled receptors, from Y1 to Y6 [14]. Its particularly high abundance is found in noradrenergic neurons, from which NPY co-released with norepinephrine (NE) is involved in the regulation of vascular tone. In the brain, this compound is engaged in feeding regulation, neuroendocrine secretion and anxiety and stress responses [15,16].

NPY has been implicated as an important regulator of inflammation, acting mainly through the NPY-Y1 receptor expressed by various immune cells to exacerbate allergic airway inflammation. The absence of NPY and NPY-Y1 receptor signaling protected mice from developing airway inflammation and delayed-type hypersensitivity [17]. These findings were confirmed in further studies in which NPY levels in bronchoalveolar fluid were elevated in an allergic model of asthma, accompanied by increased NPY expression localized to macrophages in the lung tissue. Furthermore, NPY-Y1 and -Y5 receptors were overexpressed by inflammatory and structural cells in lung tissue [18]. The critical role of NPY in the development of airway hyperresponsiveness (AHR), airway inflammation, airway dendritic cell accumulation and promotion of the type 2 immune response has been further supported [11]. The authors also demonstrated that the NPY-Y1 receptor antagonist (BIBO-3304) was able to reduce AHR and airway inflammation in wild-type mice, implying a new therapeutic avenue for the treatment of asthma.

In addition to its immunological effects on the asthmatic process, NPY has also been shown to induce direct contraction in isolated guinea-pig airways [19]. Increased NPY expression in the airway epithelium of transgenic mice was also able to induce the AHR phenotype without airway inflammation [20].

### 2.2. Neurotensin (NT)

Neurotensin is a 13 amino acid gut–brain peptide hormone, distributed throughout the central nervous system, where it is associated with dopaminergic system, regulates hypothermia, pain perception, stress responses and locomotor activity. In the periphery, neurotensin is found in enteroendocrine cells of the small intestines [21]. The pulmonary parenchyma appears to be an important site of NT metabolism, because NT concentrations increase in mixed venous blood after passage through it, both in normal and asthmatic subjects [22]. To date, there are only a few papers suggesting a potential role for this neuropeptide in asthma. Two articles from the 1990s, recounting in vitro experiments, showed that NT was a potent constrictor of airway muscles [23,24], while a later study showed the opposite effect in the form of NT-mediated inhibition of guinea pig cholinergic and noncholinergic bronchial ring contraction evoked by electrical field stimulation [25]. Consistent with a recent article about the latest in vivo study, which applied NT in non-allergic murine asthma model, showed that NT was very effective in attenuating airway hyperreactivity and inflammatory responses. Reduction in inflammatory cell number (macrophages and neutrophils) and TNF-α level in bronchoalveolar lavage fluid (BALF) and airway hyperreactivity was due to NTS1 receptor activation [10]. In the same model of asthma, a chimeric peptide constructed from modified NT and endomorphin-2 pharmacophores also reduced airway hyperresponsiveness, inflammatory cells influx in BALF, concentration of mouse mast cell protease, malondialdehyde, NF-κB expression and secretory phospholipase 2 activity in lung tissue, as well as production of pro-inflammatory cytokines in BALF and lung [26]. Some of the chimera effects depended also on NTS1 receptor stimulation [27].

### 2.3. Neuropeptide S (NPS)

NPS-expressing cells are found in amygdala, hypothalamus and pericoerulear region of the brainstem. This 20 amino acids peptide is involved in modulation of arousal and wakefulness, feeding behavior, anxiety responses and analgesia [28,29]. NPS has been implicated to have a key role in asthma pathogenesis, as a number of single nucleotide polymorphisms within NPSR1 receptor were shown to be associated with a higher genetic risk and prevalence of asthma [30,31]. In the earlier study, lung NPS receptors were reported to be upregulated in a mouse model of ovalbumin (OVA)-induced airway inflammation [32], which was confirmed by increased expression of the NPSR-B isoform in bronchiolar smooth muscle and epithelial cells of asthmatics [33]. The role of NPSR1 in childhood asthma is also indicated by higher levels of NPSR1 protein in the plasma of children with mild intermittent asthma compared to healthy controls [34]. It was further shown that NPS applied intracerebroventricularly was able to reduce airway responsiveness to methacholine in experimental animals, most likely through a CNS-mediated pathway connecting respiratory and stress responses [35]. NPS receptor has been also shown as a modulator of immune cell function. Eosinophils from patients with severe asthma and elevated serum IgE levels expressed higher levels of NPS receptor protein and responded to NPS by enhanced migration and adhesion molecule expression [36].

Despite the above studies, there is a lack of direct evidence for a role of NPS receptors in the development of asthma, especially considering that NPSR1 deletion in mice did not result in changes in allergic airway inflammation and hyperresponsiveness [35].

### 2.4. Nociceptin (N)/Orphanin (OFQ)

The heptadecapeptide nociceptin, also known as orphanin FQ, is an endogenous ligand for the nociceptin opioid-like receptor-1 (NOP1). Nociceptin and its receptor are found in central and peripheral nervous tissue, where they are involved in nociception, mood disorders, anxiety, memory regulation, food intake and immunomodulation [37]. They also have been shown to play a critical role in the pathogenesis of airway inflammation, hyperresponsiveness and bronchoconstriction [38]. With regard to the latter, nociceptin inhibited substance P release and non-adrenergic non-cholinergic contraction in guinea-pig isolated trachea [39]. D’Agostino et al. [40] demonstrated in vitro that a decrease in endogenous N/OFQ, or the absence of its receptor, results in an increase in capsaicin-induced bronchoconstriction, indicating the ability of this neuropeptide to modulate bronchoconstriction by affecting sensory fibers.

In the lungs, NOP receptors were expressed on the bronchial afferent nerve fibers of guinea pigs [41], and in human and mouse immune and structural airway cells [42]. N/OFQ immunoreactivity appeared to be increased in biopsies of asthmatic human lungs, mostly in sub-epithelial and extracellular matrix areas. Its elevated level was also found in severe asthma human sputum. The article suggested that the concentration of endogenous N/OFQ, although elevated in asthma, is too low to modulate the immune system and AHR [43]. In fact, administration of NOP receptor selective agonist UFP-112 during sensitization phase significantly reduced the AHR and lung eosinophilic infiltration in allergen-sensitized mice [44]. Subsequent research demonstrated, as well, that exogenously administered N/OFQ in experimental OVA-induced asthma reduced airway constriction and inflammation by diminishing eosinophil influx, production of Th2 cytokines and mucin [43]. Further, modulation of Th2 mediated allergic response by N/OFQ was demonstrated with its direct activity on dendritic cells [45]. Recent studies in an OVA-induced allergic asthma model have confirmed the beneficial effects of N/OFQ on disease symptoms, not only protecting against inflammation, but also against mechanical damage and remodeling of small airways [46].

### 2.5. Bombesin (BN)

Bombesin (BN), an amidated tetradecapeptide, was at first isolated from the skin of European frog [47]. Two mammalian bombesin-like peptides (BLPs) having a widespread distribution in the central nervous system (CNS) and gastrointestinal (GI) tract [47] were discovered so far: gastrin-releasing peptide (GRP), with a higher affinity towards G-protein-coupled GRP receptor, and neuromedin B (NMB) binding to the NMB receptor. The third bombesin receptor, an orphan receptor BRS-3, has no identified native ligand [48]. Moreover, mRNA for all BPLs receptors is present in mammalian airway epithelium, mucosal neuroendocrine and non-neuroendocrine cells [49,50,51]. BLPs and their receptors are suspected to be involved in developing airway hyperreactivity [52] and airway allergic disease [53,54]. It was displayed that BN and related agonists produced a potent contractile response in guinea pig peripheral airways in vitro via a direct effect on bronchial smooth muscle GRP-preferring receptor [52]. BLPs, especially BN, have proinflammatory properties; when administered into the trachea in mice or used in vitro, they induce mast cell proliferation and chemotaxis [54]. GRP activates specific signaling pathways that promote neutrophil migration, which was blocked by RC-3095, a selective GRPR antagonist [55,56,57]. GRP receptor blockade may serve as a broad spectrum of anti-inflammatory therapy for asthma [58,59], since reduced neutrophilic inflammation and cytokine production triggered by ozone were reduced with GRP blocking agent or antibody [53].

A recent investigation has revealed that BRS-3 may be a contributing factor to the pathophysiology of bronchial asthma [60]. BRS-3 activation in human bronchial epithelial cells (HBECs) was reported to promote TGF-β1 mediated activation of fibroblasts, thereby promoting the airway remodeling [61]. In contrast, another study in an animal model of asthma showed a beneficial effect of BRS-3 activation on asthma by promoting antigen uptake by HBECs and subsequently increasing T-cell proliferation and Th1 differentiation [62]. This shows another pathway of affecting asthma via stimulation of BRS-3 receptors and inhibition of Th2 inflammation response in airway mucosa.

### 2.6. Somatostatin (ST)

Somatostatin is widely expressed both in the CNS [63] and in the peripheral tissues [64,65]. Its action is mediated by five somatostatin receptor subtypes, from sst1 to sst5, which belong to the G-protein-related receptor family [66,67,68], all of which are expressed in the lung [69]. The most relevant receptor in asthma pathology appears to be sst4, whose mRNA expression has been confirmed on mouse and human bronchial epithelial, vascular endothelial and smooth muscle cells [70]. A study by Helyes et al. [71] showed that somatostatin released from sensory nerve terminals in response to activation of vanilloid 1 (TRPV1) receptors/ion channels, during lung endotoxin-induced airway inflammation, inhibited inflammation and consequent bronchial hyperreactivity. Research on the application of selective somatostatin sst4 receptor synthetic agonists in ovalbumin-induced airway inflammation has reported reduced granulocyte recruitment, infiltration of eosinophil cells, mucosal oedema formation, enhanced mucus production, destruction of the epithelial cells and AHR to carbachol. These effects can be possibly mediated via that prejunctional somatostatin sst 4 [72] receptor, whose stimulation produces the inhibition of the release of the proinflammatory neuropeptides, such as substance P and calcitonin-gene-related peptide (CGRP), from the peripheral terminals of capsaicin-sensitive sensory nerve endings [73,74]. In summary, the use of native somatostatin in the treatment of asthma is limited because of its wide range of action through all receptors, but selective agonists acting on the SST4 receptor offer some promise [65,72].

### 2.7. Vasoactive Intestinal Polypeptide (VIP)

**Figure 2:**
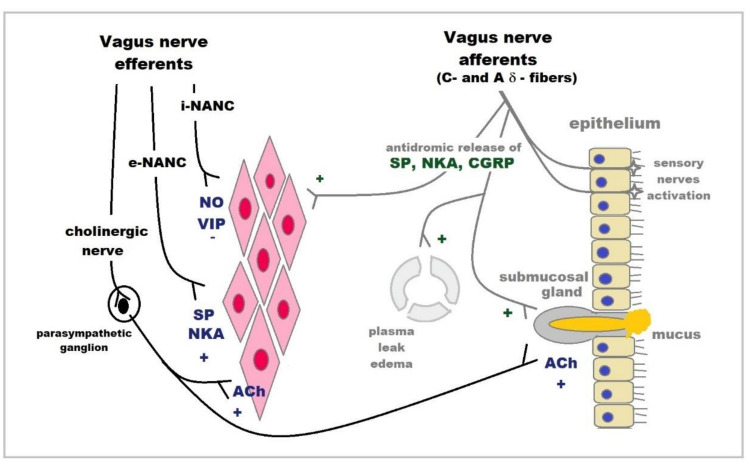
Neuropeptides secreted by i-NANC (VIP) and e-NANC (SP, NKA) efferent fibers of the vagus nerve and returning collaterals of vagus nerve sensory fibers (SP, NKA and CGRP) in the course of asthma; (−) means smooth muscle retraction and bronchodilation; (+) means smooth muscle contraction, bronchoconstriction, mucus hypersecretion, plasma exudation and edema.

Vasoactive intestinal polypeptide (VIP), a peptide messenger present in the central and peripheral nervous systems, is involved in a number of biological functions [75], including smooth muscle relaxation, regulatory hormone secretion and regulation of the immune response [76,77]. In the respiratory system, VIP-immunoreactive nerves are present in the human lung and nasal mucosa; and in the smooth muscle layer, airway glands and the walls of pulmonary and bronchial vessels [76,78,79,80,81]. VIP released from inhibitory nonadrenergic noncholinergic (i-NANC) nerves acts as a potent smooth muscle relaxant inducing bronchodilation (Figure 2) and vasodilation [82]; however, during allergic condition, VIP effects can be attenuated by released inflammatory mediators that result in AHR [83].

Although VIP is mainly secreted by nervous tissue, it is also produced by several immune cells, such as eosinophils, mast cells and lymphocytes [84,85,86]. The VIP immune response is mediated by specific receptors, namely vasoactive intestinal peptide receptor type 1 (VPAC-1) and type 2 (VPAC-2) [87]. VIP is a well-known anti-inflammatory factor that regulates the production of both anti- and pro-inflammatory mediators and the endogenous oxidant/antioxidant balance; reduces IL-1β-induced neutrophil recruitment to the airways; inhibits the activation of macrophages, dendritic cells and microglia [88,89,90]; and promotes a type 2 immune response via the VPAC-2 receptor, which is highly expressed on activated T cells and ILC2s [77,91,92]. Transgenic mice with high constitutive VPAC2 expression in T cells showed immediate-type allergic reaction, in contrast to mice with knockout of the VPAC2 receptor [91,92,93]. On the other hand, inhalation with VPAC2 agonist has been shown to induce bronchodilatation [94]. A role for VIP in the development of asthma has also been demonstrated in studies involving mice lacking the VIP gene that exhibited spontaneous asthma features, such as peribronchial airway inflammation and the production of pro-inflammatory cytokines and AHR, which was partially reduced by administration of exogenous VIP [95]. Therefore, the development of long-acting VIP analogs with strong bronchodilatory effects [76,82] or modulators of the VIP-VPAC2 signaling pathway may provide clinically useful agents for the treatment of asthma [92].

### 2.8. Tachykinins

Tachykinin peptides are one of the largest family of neuropeptides present from invertebrates to mammals. They are derived from the posttranslational processing of three preprotachykinin precursors: preprotachykinin A, consisting of substance P (SP), neurokinin A (NKA), neuropeptide K (NPK) and neuropeptide-γ (NPγ); preprotachykinin B—neurokinin B (NKB) [96]; and several related peptides in different species, such as hemokinin-1 (HK-1) in mice, endokinin-1 and -2 (EK-1 and EK-2) in rabbits, and endokinins A–D (EKA, EKB, EKC and EKD) in humans [97]. All tachykinins share common C-terminal sequence and affinity to all tachykinin receptor with a various potency (for details, see Table 2). A case-control study in the Canary Islands revealed a significant association between tachykinin gene polymorphism and asthma [98].

There are two sources of tachykinins in the airways: neural and non-neural. The main sources of SP and NKA are considered to be not only sensitive to capsaicin primary afferent neurons [104,105] but also excitatory nonadrenergic noncholinergic (e-NANC) nerves [106,107] (Figure 2). Substance P and NKA were also detected in neurons within the epithelium, around blood vessels and submucosal glands and within the bronchial smooth muscle layer [105,108]. Non-neuronal sources of tachykinins in the airways were reported in epithelium (SP) [109] and airway smooth muscle [110]. Moreover, SP was shown to be produced by eosinophils, monocytes, macrophages, lymphocytes and dendritic cells [111,112,113,114]. NKB was not detected in the airways so far [115]. Classical tachykinins (SP, NKA and NKB) possess a wide distribution in the central and peripheral nervous system, which is one of the major sources of these peptides [116]; however, HK-1 is predominantly expressed in non-neuronal tissues as indicated by Page and coworkers [99] in the lung, bone marrow, thymus, skeletal muscle and lymph node, as well as together with SP by various inflammatory and immune cells, such as T and B lymphocytes, macrophages and dendritic cells [117,118]. Tachykinins are involved in a cough reflex and in the inflammatory response in the lungs, as they were shown to be important mediators of the neurogenic inflammation in asthma, allergic rhinitis and chronic bronchitis [119]. Substance P for instance acts directly on airway goblet cells, as well as submucosal glands inducing mucus secretion [120,121]. SP and HK-1 generated from macrophages, bronchial cells and mast cells cause degranulation of human mast cells via MRGPRX2, Mas-related G protein-coupled receptor-X2, which is upregulated in the lung mast cells and serum of asthmatic patients [122]. SP has been shown to enhance TNF-alpha synthesis and secretion from human mast cells [100]. Endogenous tachykinins have been shown to also modulate the IL-17-induced neutrophil recruitment in vivo in rat airways. The selective blockade of NK1, in contrast to blockade of NK2 receptors, limits the recruitment of neutrophils in rat airways [101]. There is also strong evidence indicating the role of tachykinins and tachykinin receptors NK1 in recruiting eosinophils into the airways after allergen challenge [119]. Although the SP content in the sputum or BAL of asthmatics is elevated compared to healthy controls, it seems to have minor effects on the acute phase of allergic asthma. SP applied intravenously or via inhalation in human subjects was not able to produce bronchoconstriction in contrast to NKA [123], although a role for tachykinins in muscle contraction is indicated by the presence of all NK1, NK2 and NK3 receptors in human airway smooth muscle [102]. However, both peptides SP and NKA have been shown to be involved in the development of airway hyperreactivity (Table 3) [119]. In contrast to SP and NKA—both being “classic tachykinins”—HK-1 had no influence over bronchial hyperreactivity [124]. Application via inhalation of CS-003, a triple tachykinin receptors antagonist, evoked diminished bronchoconstriction after methacholine provocation [103]. On the other hand, there is also a less optimistic study showing that chronic administration of an NK1 receptor antagonist, maropitant, was ineffective in reducing signs of neurogenic airway inflammation, eosinophilia and AHR in an experimental feline model of asthma [125]. The more detailed information on the role of tachykinins in asthma was described in the latest review by Pavon-Romero et al. [5]

### 2.9. CGRP

Although the calcitonin gene–related peptide (CGRP) has been described quite thoroughly in the context of its role in asthma in the article by Pavon-Romeiro et al. [5], it is impossible not to mention it in this review. CGRP is synthesized in the airway epithelium, in neuroepithelial bodies and together with SP in the sensory nerve endings of the vagus C-fiber and released into the airway when an allergic stimulus is acted upon (Figure 2) [126,127]. It was established that CGRP is involved in the late asthmatic response, evoking vasodilatation, mucus secretion and edema in the airways [128,129,130]. Although CGRP has been described to potentiate tracheal muscle contraction in response to capsaicin and an electric field stimulation in vitro [131,132], a more recent study has shown that exogenous CGRP in an allergic mouse model of asthma was able to reduce AHR and eosinophilic inflammation [133]. As it turns out, the role of this peptide in the pathogenesis of asthma is far from clear. A further study showed that the transfer of CGRP-pretreated dendritic cells to in vivo model of allergic asthma reduced airway inflammation, shown as decreased BALF eosinophil influx and increased IL-10 concentration [134].

## 3. Adipokines

### 3.1. Adiponectin

Adiponectin participates in the regulation of energy metabolism at the level of adipose tissue and the liver [135] and sensitizes body cells to insulin [136]. Adiponectin receptors and transport molecules are expressed in the lungs [137,138,139], where adiponectin has been shown to reduce pulmonary inflammation [140,141]. The overall anti-inflammatory action of adiponectin involves the inhibition of NF-𝜅B activation and production pro-inflammatory cytokine IL-6 and the induction of the expression of anti-inflammatory IL-10 [142,143].

There are contradictory data about the relationship between adiponectin and asthma in the general human population. It seems that its action depends not only on the sex and age of the participants, but also on their hormonal status (pre- and post-puberty; pre- and post-menopause) and the presence and degree of obesity. Lower levels of adiponectin have been observed in asthmatics [144,145,146,147,148,149]. In general, low adiponectin levels are associated with a higher incidence of asthma [115], poor lung function [150,151,152,153] and increased risk of asthma [154,155]. At the same time, higher adiponectin levels seem to play a protective role [156]. In this context, Ding et al. [157] found that, during exacerbations, adiponectin levels significantly decreased, together with an increase of inflammatory markers, such as like IL-6 and TNF-α, indicating that adiponectin may play a protective role in the pathogenesis of asthma.

In contrast, other researchers [158,159,160] could not find any correlation between adiponectin, asthma and lung function. In animal models of OVA-induced asthma, Nigro et al. [161] found decreased levels of adiponectin. At the same time, in OVA-sensitized mice, continuous administration of exogenous adiponectin alleviated symptoms of the disease (Table 4) [162]. More research data about adiponectin, its mechanisms of action and its role in asthma were recently presented in an excellent review by Otelea et al. [163].

### 3.2. Leptin

Leptin is an energy-regulating adipokine released in response to feeding and inflammation or infection [164,165]. It plays an important role not only in energy metabolism but also in learning processes [166]; hormonal changes, fertility, onset of puberty and regulation of bone mass [167]; and immunity [168,169], including inflammatory disorders of the respiratory system. Leptin is expressed by various cell types of the respiratory system including bronchial and alveolar epithelial and smooth muscle cells, macrophages and bronchial submucosa, and, as a consequence, it can be found in BALF [169,170,171,172]. Leptin is a pro-inflammatory adipokine and is believed to promote pulmonary inflammation and bronchoconstriction [145].

Most of the studies show that asthmatic subjects have higher leptin levels [132,144,145,151,159,160,173,174,175,176,177,178,179]. Higher leptin levels are believed to be associated with asthma severity [180], lower lung function [176,181] and airway hyperreactivity [151]. Leptin levels may be an indicator of asthma control, as its levels rise during exacerbations in adults [182,183]. Moreover, a high leptin level increases the risk of developing asthma [184,185]. Some reports failed to find any association between leptin levels and asthma, its incidence and severity [186,187,188,189,190,191]. One has to keep in mind that leptin takes part in the pathophysiology of both asthma and obesity and that outcomes of these diseases may overlap, especially in the context of lung function [156,192,193,194].

The pro-inflammatory effect of leptin has been proven in animal models by Shore et al. [195,196], Lu et al. [197] and Johnston et al. [198], who found that leptin administration enhances airway inflammation. As it has been presented by Shore et al. [196], leptin levels are not only increased in asthma, but leptin itself increases airway hyperreactivity and pro-inflammatory cytokines levels in BALF when administered during sensitization in the OVA allergic asthma model in mice [196]. This suggests that leptin is released in response to inflammation and that leptin itself enhances inflammation [168,195,196,199].

### 3.3. Resistin and Resistin-like Molecule Family (RELM) Proteins

Resistin is a member of the resistin-like molecule family (RELM) and was previously known as “found in inflammatory zone”. It is secreted by the adipose tissue, and its levels are increased in diet-induced and genetic forms of obesity [200].

Resistin is associated with inflammatory diseases [201], including those of the lungs [202,203]. It is expressed in airway epithelial cells and other cells of the respiratory system [204], where it increases the expression of various mucins [205]. In general, this peptide shows pro-inflammatory activity; when administered to macrophages, resistin induces the release of pro-inflammatory cytokines, including TNF-α [206], which in turn enhances the release of resistin [207]. Its pro-inflammatory action occurs mostly via various signaling pathways, including NFκB [206]. It seems to act in contrast to adiponectin, which was reported to inhibit the effects of resistin [208]. The role of resistin in immunity and inflammatory diseases has been summarized in detail elsewhere [204,209,210]. RELMs are believed to display inflammation-regulating, chemokine and growth-factor properties [204] and are involved in propagation of oxidative stress [211]. While RELMα seems to have an important role in inflammation and airway remodeling in rodents [212,213,214,215,216,217], and its overexpression seems to protect from development of asthma symptoms in mice [216], the influence of RELMβ on asthma outcomes is ambiguous [203,218]. Pine et al. [219] sum up findings on the role of RELMα/β and resistin in signaling and inflammatory disorders, showing differences between these peptides. Together with Fan et al. [220], they point to slightly different properties of members of the RELM family; their actions seem to vary between rodents and humans, as well as between asthma models.

**Figure 3:**
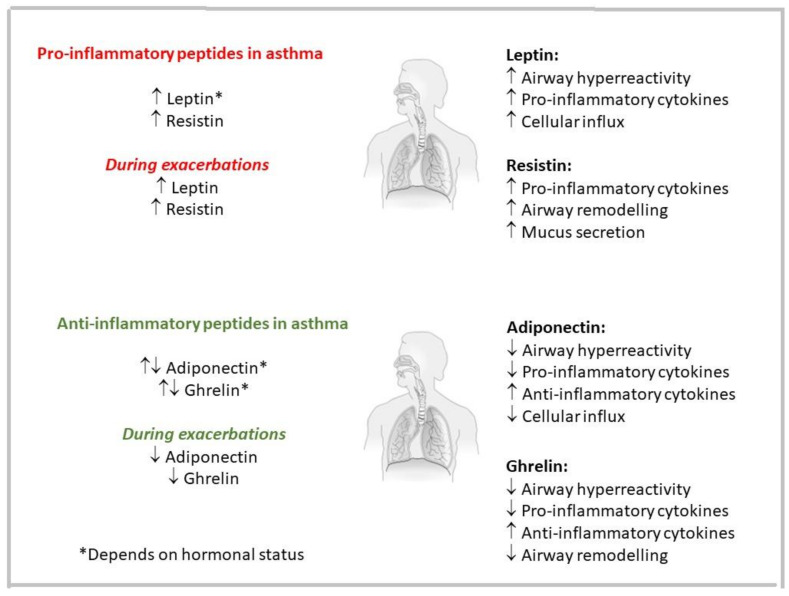
Influence of pro- and anti-inflammatory obesity-related peptides on the main features of asthma. One must keep in mind that it is not the concentration of the respective peptide alone that counts but also the ratios between them [135]. * is present in the figure body.

Resistin levels are higher during asthma exacerbations, as compared to healthy controls, but not during their resolution or follow-up [183]. Additionally, Al Mutaivi et al. [221] observed higher resistin levels in asthmatics, together with an inverse correlation of its concentration with lung function, as did Ballantyne et al. [222], Vezir et al. [223] and LaRochelle et al. [202], who found a link between plasma resistin concentrations and asthma severity. As a consequence, resistin could be a predictor of asthma risk and control. Fang et al. [214,215] and Grainge et al. [224] observed higher expression of RELMβ in asthmatic humans, which correlated inversely with lung function and positively with mucin production. RELMs and resistin may be involved in airway remodeling and asthma progression [213]. In contrast, Kim et al. [189] found lower resistin levels in asthmatic minors compared to healthy ones and pointed to resistin levels as negative predictors of asthma. As for other adipokines, differences between adults and children might be due to a different hormonal status of both groups also at the level of adipokines. Once again, as is the case of all adipokines, the level of the respective peptide is not as important as the ratios between all of them (Figure 3) [135]. Another interesting resistin issue was described by Leivo-Korpela et al. [180], who found no difference in resistin levels between non-obese female asthmatics and a matched control group after adjusting for BMI. However, higher resistin concentrations predicted a favorable response to inhaled corticosteroids.

## 4. Stomach Peptide

### Ghrelin

Gastrin-associated ghrelin is a peptide involved in the regulation of energy metabolism and plays a possible role in the pathogenesis of asthma. It is released from the stomach in response to hunger and, thus, regulates the food intake [228]. As is the case with adipokines, its levels are disturbed in obesity and metabolic disorders. Ghrelin is expressed in many tissues, including lungs and bronchial epithelial cells [229,230], and its receptors have been found in lung parenchyma [231]. It is believed to have antimicrobial and anti-inflammatory properties [232,233], mostly by downregulation of expression of pro-inflammatory cytokines and upregulation of the anti-inflammatory ones both in vitro and in vivo [232,234,235,236]. Moreover, ghrelin attenuates neutrophil migration [225]; decreases fibroblast activity, preventing organ fibrosis [237]; and decreases collagen production [238]. Its cytoprotective properties rely mostly on the suppression of endoplasmic reticulum-related stress [239].

The anti-inflammatory action of ghrelin occurs mostly via the inhibition of the NFκB pathway [240,241], and not by scavenging intracellular ROS [226] even if ghrelin has antioxidant properties [228,233,242]. The influence of ghrelin on oxidative stress inflammation and its role in immunity has been summarized by Wang et al. [243], Baatar et al. [225] and Jafari et al. [244], among others, who detailed the characteristics of the cytokines involved in the anti-inflammatory action of ghrelin.

Up to now, there is only one report on the influence of ghrelin on asthma symptoms in animals. In OVA-sensitized mice, ghrelin administration decreased almost all asthma-related features, including cellular influx into the airways, pro-inflammatory cytokine levels in BALF, thickening of the airway epithelium and airway hyperreactivity [227]. As for humans, observations regarding ghrelin levels in asthma and its potential role are conflicting. Matsumoto et al. [147], Tsaroucha et al. [245] and Yuksel et al. [246] found lower ghrelin levels in asthmatics as compared to controls, but no correlation between ghrelin levels and asthma severity. Moreover, lower ghrelin levels have been found during exacerbations than during stable periods in the same subjects [245]. In addition, Matsuda et al. [247] described how ghrelin levels negatively correlated with IgE production, leading to the conclusion that ghrelin might inhibit directly or indirectly IgE synthesis. In contrast to the abovementioned research, Al-Ayed et al. [248] found higher ghrelin levels in asthmatic children than in non-asthmatic ones. Moreover, subjects with uncontrolled asthma had higher ghrelin than patients with well-controlled disease. Higher ghrelin levels were also found in asthmatic adults by Toru et al. [249].

In chronic respiratory infections in undernourished patients, Kodama et al. [250] found that ghrelin administration suppressed airway inflammation, decreased accumulation of neutrophils in the lung and led to weight gain. Miki et al. [251] described an increased respiratory strength and better respiratory outcomes of COPD patients after ghrelin treatment, thus indicating a possible therapeutic role of ghrelin in other obstructive and inflammatory diseases of the respiratory system, such as asthma.

## 5. Antimicrobial Peptides (AMPs)

Another group of peptides employed by the host to discard microorganisms are antimicrobial peptides, presenting diverse biological functions. One of them is LL-37, a 37 amino acid cationic peptide belonging to the cathelicidin family of antimicrobial peptides [252,253]. The most important factors that stimulate the production of LL-37 are pathogens (particles of bacterial origin), skin damage and vitamin D3. They stimulate LL-37 expression in epithelium and inflammatory cells [254], where, apart from antimicrobial activity, it is involved in wound healing, angiogenesis, cell apoptosis and immunomodulation [255]. The impact of LL-37 on allergic asthma seems to be rather proinflammatory (Table 5), since it is chemotactic to eosinophils, neutrophils and mast cells and deteriorates airway inflammation and AHR in a mouse model of allergic asthma [255]. What is more, LL-37 has been displayed to induce eosinophilic release of cysteinyl leukotrienes, which are potent bronchoconstrictors, also exacerbating inflammation [256]. It is not without reason that bacterial infections, which induce the release of LL-37, account for approximately 50% of asthma exacerbations [257].

The largest group of AMPs produced by mammals are defensins, first discovered in human neutrophils. In humans, α- and β-defensins are found in neutrophil granules, macrophages, NK cells, skin, body fluids and respiratory mucosa, among others [252]. Human β-defensin 1 (hBD-1) is expressed constitutively in the respiratory tract, while β-defensin 2 (hBD-2), hBD-3 and hBD-4 are induced by infectious agents, tissue damage or proinflammatory cytokines [254]. Defensins are chemoattractants for immune cells; they also activate dendritic cells and mast cells to degranulate, resulting in the release of histamine and other inflammatory and allergic mediators [254]. Defensins, which act as inflammatory mediators, appear to be involved in the pathogenesis of asthma. Associations between asthma diagnosis and genetic variation in human hBD-1 encoding gene have been suggested [258]. Borchers et al. [259] hypothesized that variants in the gene encoding hBD-2 may be a risk factor for the development of asthma if they result in insufficient production of hBD-2. As confirmation, multiple genetic variants of this gene have been identified associated with asthma and allergy in the pediatric population. Additionally, it was confirmed in a mouse model of asthma that the application of exogenous hBD-2 prevents key symptoms of asthma, such as BALF inflammatory cell influx, cytokine production and AHR [259,260]. This suggests that hBD-2 has a mitigating effect on asthma symptoms.

## 6. Conclusions

The plethora of described peptides involved in the regulation of asthma-specific symptoms suggests many opportunities to explore new therapeutic pathways for this complex disease. Their advantage is that they are natural endogenous peptides present in the human body. However, sometimes the multiplicity of receptors and bodily functions they can affect prevents their use as potential therapeutics. The solution may be the search for new selective agonists or antagonists. Some of the studies described in the review based on animal models so far have shown beneficial effects in reducing asthma symptoms by activating nociception NOP1, bombesin BRS-3 and somatostatin SST4 receptors, or blocking the NPY-Y1 receptor. Peptides related to energy, glucose metabolism and turnover play an important role in the pathophysiology of obesity-related asthma. They can enhance (e.g., leptin or resistin) or alleviate (e.g., adiponectin and ghrelin) various features of asthma, acting either pro- or anti-inflammatory, respectively. Despite the strong interest in peptides, further studies exploring their true role in asthma pathology are needed. Sometimes this role is still unclear, and it is not uncommon for opposite data to be published.

## Figures and Tables

**Table 1:** Regulatory peptides involved in mediating asthma symptoms. A description of the action of a particular peptide and the receptors through which it acts (animal studies).

Peptide (Sequence)	Asthma Suppression	Asthma Promoting	Receptors	References
Neuropeptide Y (NPY) (YPSKPDNPGEDAPAEDLARYYSALRHYINLITRQRY)		Induction of AHR		[19,20]
		↑ AHR and airway inflammation	NPY-Y1	[11,19]
Neurotensin (NT) (XLYENKPRRPYIL)	↓ AHR and inflammation		NTSR1	[10,26,27]
Neuropeptide S (NPS) (SFRNGVGTGMKKTSFQRAKS)	↓ AHR			[35]
Nociceptin (N)/Orphanin OFQ (FGGFTGARKSARKLANQ)	↓ airway constriction ↓ inflammation		NOP1	[40,43,44,46]
Bombesin (BN) (XQRLGNQWAVGHLM)		↑ neutrophilic inflammation ↑ cytokine production	GRPR	[53,55,56,57]
		↑ contractile response	GRPR	[52]
		↑ mast cell proliferation and chemotaxis		[54]
		↑ airway injury and re-modeling	BRS-3	[58,61]
**Somatostatin (ST)** (AGCKNFFWKTFTSC)	↓ inflammation and AHR		sst4	[71,72,73,74]

**Table 2:** Alignment of the amino acid sequence of the mammalian tachykinin and their preferences for tachykinin receptors [99]. The role of NKA receptors in asthma.

Peptide (Sequence)	Affinity [99]	Activation of NKA Receptors	
SP (RPKPQQFFGLM)	NK1 > NK2 >> NK3	NK1 ↑ inflammatory cells recruitment [100,101]	NK1, NK2, NK3 ↑ airway smooth muscle constriction [102,103]
NKA (HKTDSFVGLM)	NK2 > NK 3> NK1		
NKB (DMHDFFVGLM)	NK3 > NK2		
HK1 (RSRTRQFYGLM)	NK1 >> NK2 >> NK3		
Endokinin (A/B GKASQFFGLM)	NK1 > NK2 >> NK3		
Endokinin C (KKAYQLEHTFQGLL)	NK3		
Endokinin D (VGAYQLEHTFQGLL)	NK3		

**Table 3:** Neuropeptides released by vagus efferents (i-NANC, e-NANC) and retrogradely released by afferents (capsaicin C-fibers) contributing to asthma symptoms. A description of the action of a particular peptide and its receptor involved.

Peptide (Sequence)		Asthma Suppression	Asthma Promoting	Receptors	References
Vasoactive intestinal polypeptide (VIP) (HSDAVFTDNYTRLRKQMAVKKYLNSILN)		Mice lacking the VIP gene exhibited spontaneous asthma features including AHR			[95]
		Bronchodilatation		VAPC2	[87,94]
TACHYKININS	Substance P (SP) (RPKPQQFFGLM)		↑ mucus secretion ↑ development of AHR↑ recruitment of airwayneutrophils, ↑ degranulation of mast cells	NK1	[100,101,119,122]
	Neurokinin A (NKA) (HKTDSFVGLM)		↑ bronchoconstriction ↑ development of AHR	NK2	[102,119,123]
	HK-1 (RSRTRQFYGLM)		↑degranulation of mast cells	NK-1, MRGPRX2	[122]
Calcitonin gene–related peptide (CGRP) (ACDTATCVTHRLAGLLSRSGGVVKNNFVPTNVGSKAF)			↑ vasodilatation, ↑ mucus secretion and edema ↑ trachea muscle contractions to capsaicin and electrical field	RAMP 1	[5,128,129,130,131,132]
		↓ AHR and eosinophilic inflammation			[133]

**Table 4:** Obesity-related peptides involved in mediating asthma symptoms. A description of the action of a particular peptide and the receptors through which it acts.

Peptide	Asthma Suppression	Asthma Promoting	Receptors	References
Adiponectin	Reduces airway inflammation		T-cadherin	[140,141,147]
Leptin		Promotes airway inflammation and bronchoconstriction	Ob-R	[145,169,194]
Resistin		Promotes airway inflammation and airway remodeling	Not known	[204,209,210]
Ghrelin	Reduces airway inflammation and airway hyperreactivity		GHS-R	[225,226,227]

**Table 5:** Description of the effect of antimicrobial peptides on asthma pathology.

Peptide	Asthma Suppression	Asthma Promoting	References
LL-37		Proinflammatory and exacerbates inflammation and AHR	[254,255,256]
β-defensin 1 (hBD-1)		Promotes airway inflammation; chemoattractant for immune cells	[254,258]
β-defensin 2 (hBD-2)	Prevents airway inflammation and AHR		[259,260]
